# Ground reaction force as a factor responsible for the topography of injuries in professional dance. An analysis of three dance styles: classical dance, modern dance, and folk dance

**DOI:** 10.5271/sjweh.4137

**Published:** 2024-03-01

**Authors:** Joanna Gorwa, Katarzyna Nowakowska-Lipiec, Robert Michnik

**Affiliations:** 1 Department of Biomechanics, Faculty of Sport Sciences, Poznan University of Physical Education, Poznań, Poland.; 2 Department of Biomechatronics, Faculty of Biomedical Engineering, Silesian University of Technology, Zabrze, Poland.

**Keywords:** ballet dancer’s load, biomechanics of dance, dancer, impact force, occupational medicine, performing artist

## Abstract

**Objective:**

The study aimed to identify the effects of ground reaction forces (GRF) recorded during landing in typical elements of three dance styles, including classical, modern, and folk dance, on injuries' topography.

**Methods:**

The research involved a survey and measurements of GRF generated during landing after the jump. The survey involved a group of 90 professional dancers. In the questionnaire, the dancers marked areas of the human body that were affected at least once by injuries. Biomechanical tests of the GRF recording were conducted on a group of 15 professional dancers. The analysis focused on the following parameters: a maximum value of the vertical variable of the GRF relative to body weight (maxGRF_z_), the time between the moment from first foot contact with the ground to the moment of reaching the maxGRF_z_ (t_maxGRFz_), and the loading rate of the GRF relative to body weight (LR_GRFz_).

**Results:**

Regardless of dance style and sex, the lower spine, knee joints, ankle joints and feet were the areas most affected by injuries among professional dancers. The level of maxGRF_z_, t_maxGRFz_ and LR_GRFz_ during typical jumps in classical, modern, and folk dance was statistically significantly different (P<0.01*). The highest mean maxGRF_z_ values were recorded for jumps performed by classical dancers. Furthermore, the sum of injury-affected areas differed significantly across various dance styles and was connected with the impact forces transferred by the dancer’s musculoskeletal system.

**Conclusion:**

The level of GRF is one of the decisive factors affecting the topography of professional dance injuries.

At the beginning of the 1970s, the study of Nicholas et al (1) reported a surprising level of dynamic loads in professional dance. According to this research team, professional dance is at the top of the list of the most demanding physical activities for the musculoskeletal system, alongside American football. The analysis of studies by other authors indicates that most injuries in the professional dancer community are chronic, such as inflammation of soft issues, fatigue injuries, and pulled and torn muscles, with fractures accounting for the smallest percentage of injuries (2–4). Studies dealing with the problem of professional dancers' injuries largely agree that the feet, ankle, knee, hip joints, and spine are the body parts injured most often. On the other hand, opinions about the frequency and causes of the injuries vary among researchers (3–18).

With intensive daily use of the musculoskeletal system and a high level of impact loads generated during ballet jumps – which can be many times the body weight (BW) – permanent damage, deformations, and weakening of tissue structures occur (5, 19–21). Professional dance requires significant efforts (both long- and short-term), perfect mastering of motor activity, and technical precision, which combine to cause joint and ligament system overloads (3). It seems that the lack of recovery time, artists’ limited awareness of the impact loads they are subject to in dancing, and extremely difficult choreographies, make this professional group susceptible to injuries. One of the biggest challenges in professional dance is maintaining an active career for as long as possible. As a result, dancers need to identify which parts of their musculoskeletal system are most likely to develop health issues.

Although the issue of injuries in professional ballet dancing has been covered thoroughly, there are very few studies directly connecting the issues outlined above with the work environment and risk factors. In terms of biomechanics, such factors undoubtedly include repetitions of movement schemes and the level of impact forces affecting artists.

The most frequently investigated professional dance style is classical, yet epidemiological tests of dancers’ injuries are inconsistent (18). However, professional dancers, defined as artists for whom dance is their only source of income, are employed on a contractual basis (22) and may also perform dance styles other than classical, such as modern dance, jazz, or folk dance.

This study aimed to identify the effect of ground reaction force (GRF) values during landing in three typical dance styles – classical, modern, and folk dance – on the topography of injuries by identifying the affected areas.

## Methods

The study involved a group of professional classical, modern, and folk dancers. To be considered a professional dancer for the study, participants had to have a minimum experience of ten years (total duration of dance-related training since childhood), be employed as a dancer in a dance theatre, and earn their living solely from dance.

The study included two stages of investigation: (i) a survey and (ii) GRF measurements generated during the performance of elements of the three dance styles characteristic of the dance groups participating in the study-related tests.

All participants agreed to take part in the tests. The Bioethical Committee at the Poznan University of Medical Sciences (Poland) approved the study design before the commencement of the study (decision no. 796/09).

### Survey

Data was collected from 90 professional dancers to determine the topography of injuries, including 30 classical dancers (15 men and 15 women), 30 modern dancers (15 men and 15 women), and 30 folk dancers (15 men and 15 women, and analyzed. Table 1 presents the basic anthropometric and professional data of the artists.

**Table 1:** Somatic and professional characteristics of the studied artists by sex and dance style. [SD=standard deviation.]

	Classical dancers						Modern dancers N=30						Folk dancers N=30				
	Total (N=30)		Women (N=15)		Men (N=15)		Total (N=30)		Women (N=15)		Men (N=15)		Total (N=30)		Women (N=15)		Men (N=15)
	Mean (SD)		Mean (SD)		Mean (SD)		Mean (SD)		Mean (SD)		Mean (SD)		Mean (SD)		Mean (SD)		Mean (SD)
Age (years)	23.3 (3.7)		23.6 (3.6)		23.1 (3.9)		30.7 (7.8)		33.1 (8.5)		28.3 (6.5)		30.9 (7.7)		29.7 (7.4)		32.1 (8.1)
Body mass (kg)	62.3 (11)		53.5 (4.9)		71.2 (7.5)		65.4 (12.2)		54.9 (5.7)		75.9 (6.4)		65.4 (12.2)		54.9 (5.7)		75.9 (6.4)
Body height (m)	1.77 (0.09)		1.71 (0.05)		1.84 (0.09)		1.72 (0.09)		1.65 (0.04)		1.80 (0.05)		1.72 (0.09)		1.65 (0.06)		1.79 (0.06)
Body mass index (kg/m^2^)	19.72 (1.7)		18.3 (1.0)		21.1 (1.1)		21.89 (2.9)		20.2 (2.4)		23.6 (2.2)		21.8 (2.3)		20.0 (1.3)		23.6 (1.5)
Total experience (years)	13.2 (3.5)		12.3 (2.7)		14.1 (4.1)		18.4 (6.7)		21.6 (6.2)		15.3 (5.6)		19.3 (6.90)		19.4 (7.7)		19.3 (6.4)

The study used a questionnaire that included a diagram of the human body on which dancers marked areas where dance-related injuries occurred at least once in their career that were temporary and excluded them from training (23). A modified diagram of the human body proposed by Arnheim was used (24). It should be noted that in this part of the questionnaire, the type of injury, such as dislocation, tearing, and pulling, or the injured structure (muscle and bone), did not have to be specified, and only the area subject to overstrain was determined. The selectable areas included the Achilles tendon, ankle joint, metatarsus, phalanges, lumbosacral spine, knee joint, shank, thigh, and hip joint. If the diagram used in the survey did not contain a given body area affected by an injury in the past, such as another spine section, the dancer could add such an area. The questionnaire was completed on a voluntary and anonymous basis.

Assessment of the survey data employed a zero-one method, with a value of one attributed to a body area if it had ever been affected by an injury and a score of zero given to unaffected areas. The results provided a detailed topography of commonly injured body areas in professional dancers from distinct dance groups. Data were expressed as a percentage, and the analysis also involved calculating the mean number of areas affected by injuries in each group.

### Measurement of ground reaction forces generated during the performance of elements of three dance styles

From the group of 90 dancers who were surveyed, 15 dancers were selected for biomechanical research. Biomechanical tests aimed at determining impact forces were conducted on the dancers: 7 women with a mean age of 28 [standard deviation (SD) 6] years with a mean BW of 53.4 (SD 4.4) kg and a mean height of 166 (SD 5) cm and 8 men aged 28 (SD 5) years, weighing 72.2 (SD 7.1) kg, and with a mean height of 181 (SD 6) cm. All 15 participants were first dancers – prima ballerina, principal dancer, or first soloist – from classical, modern and folk dance backgrounds (N=5 per group). All dancers were healthy, did not suffer from chronic disease, and had not suffered an injury within the six months preceding the study.

The biomechanical tests involved GRF recordings following the performance of jumps typical of each dance style. Choreographers selected the elements based on the typical stage tasks of each dance style and the characteristics of the dance group’s repertoires. A dance teacher and an experienced biomechanical specialist in dance and occupational medicine attended the tests.

The folk dancers participating in the study dance Polish folk dances in pairs. Most of the choreographic arrangements on stage involve the performance of elements in pairs (jumps, turns, lifts). However, as in classical and modern dance, some stage elements are also performed solo. The biomechanical study included elements performed individually. All of the jumps began with a run-up and flight phase and ended with the single limb landing phase. Jumps on the measurement platform were performed barefoot.

The principal dancers performed the following style-specific jumps: (i) classical dance: grand pas de chat; grand pas assemble; entrelace; grand jeté; saut de basque; pas jeté; turn; jeté en tournant; ballonne; pas echappe, sissonne ouverte, jeté passe, and pas de poisson; (ii) modern dance: stag jump, grand jeté modern, skip with throwing leg to the side, travelling leap, jump with “ront” & twist, skip with forward leg throw, grand jeté en tournant, skip, leap, sisonne overt parallel, flat pas de chat, vertical jump by Ewa Wycichowska, vertical jump head tilted back, half stag jump, wide open legs jump to the side, flick-jeté jump, and flick-jeté leap; and (iii) folk dance (Polish folk dances, the proper names of the analysed dance jumps are given): Podcinane – Śmigło, Miotły, Skoki w obrocie (kołomajki), Skoki w sarenkach, Łamańce-tańce góralskie, Podskok przez nogę, Przeskok przez ciupagę, Araby, Kabriol Krakowski, Roznóżki, Wyrzut skoki góralskie, Kołomajki obroty, and Kabriol krakowski.

Dancers of all styles performed a total of 43 types of jumps. In each dance style, 13–17 types of jumps were analyzed, which the choreographers and dancers identified as the most frequently performed and characteristic of the style. The dancer performed each jump type three times. There was a rest period of five minutes between repetitions. No dancers performed more than 15 jumps in a single session, and different types of jumps were recorded over several measurement sessions.

Measurement of the vertical GRF component (GRF_z_) and other variables (presented below) over time utilized a KISTLER 9261A triaxial piezoelectric platform (Kistler Group, Winterthur, Switzerland) combined with a personal computer via a 12-bit and 16-channel AMBEX analog card, at a sampling rate of 1000 Hz.

The time courses of the vertical component of GRF [N] has been derived from the tests performed on the the dynamometer platform. The recorded GRFz values were normalized to the participant’s weight [BW]. Then, the following parameters were determined: (i) maxGRF_z_ [BW] - the maximum value of the GRF vertical variable relative to the weight of the participant; (ii) t_maxGRFz_ [s] - the time between the moment of first foot contact with the ground until reaching maxGRFz; and (iii) LR_GRFz_ [BW/s] – the loading rate of the GRF_z_ relative to the weight of the test subject (21).

The parameters mentioned above were determined for each of the jumps performed. The mean values were then calculated for each of the dance styles (the average of all the maxGRFz obtained during the performance of the choreographic elements by all the dancers who represent a given style of dance): classical, modern and folk dance. The most loaded style-specific jumps for each style were also reported in the paper.

### Statistical analysis

All calculations employed Statistica 13.1 software (Dell Inc, Tulsa, OK, USA) or PQStat 1.8 software (PQStat Software, Poznań, Poland). The Shapiro-Wilk test assessed and verified the normality of data distribution, and the Kruskal-Wallis one-way analysis of variance (ANOVA) compared differences between the three groups. Pearson’s linear correlation coefficient or Spearman’s rank correlation coefficient was used to test the degree of correlation between the quantitative variables analyzed (ie, number of areas affected by injuries and age, weight, total experience). Quantitative variables for the measurements of GRF_z_ were described using the mean value, SD, median, minimum, and maximum values. The survey results were listed in tables as percentages of injuries in each anatomical location for individual dance styles and sex, and the number of areas affected by injury were presented using box plots, also divided by sex and style. The level of significance adopted for statistical analyses was P=0.05.

## Results

### Survey results (body diagram results)

Table 2 presents the topography of injuries determined from the body diagram used in the questionnaire. The results are presented by sex and dance style. The survey results revealed that Achilles tendon injury prevalence was similar in males representing the three dance styles, whereas it was lower in female folk dancers. The lowest prevalence (not exceeding 30%) of ankle joint injury was observed in modern dancers, while more than 80% of classical and folk dancers experienced such an injury, irrespective of sex.

**Table 2:** Detailed topography of the most commonly injured areas of the body in professional dancers by sex and dance style. [r=result for the number of dancers indicating a given injury in the questionnaire; n=number of the group of dancers subjected to analysis. ]

Body part	Classical dancers						Modern dancers						Folk dancers				
	Total (N=30)		Women (N=15)		Men (N=15)		Total (N=30)		Women (N=15)		Men (N=15)		Total (N=30)		Women (N=15)		Men (N=15)
	% (r/n)		% (r/n)		% (r/n)		% (r/n)		% (r/n)		% (r/n)		% (r/n)		% (r/n)		% (r/n)
Achilles tendon	53.3 (16/30)		53.3 (8/15)		53.3 (8/15)		46.7 (14/30)		46.7 (7/15)		46.7 (7/15)		36.7 (11/30)		26.7 (4/15)		46.7 (7/15)
Ankle joint	83.3 (25/30)		93.3 (14/15)		73.3 (11/15)		26.7 (8/30)		20.0 (3/15)		33.3 (5/15)		83.3 (25/30)		80.0 (12/15)		86.7 (13/15)
Metatarsus	80.0 (24/30)		80.0 (12/15)		80.0 (12/15)		16.6 (5/30)		0.0 (0/15)		33.3 (5/15)		16.7 (5/30)		20.0 (3/15)		13.3 (2/15)
Foot phalanges	63.3 (19/30)		60.0 (9/15)		66.7 (10/15)		23.3 (7/30)		20.0 (3/15)		26.7 (4/15)		20.0 (6/30)		26.6 (4/15)		13.3 (2/15)
Lumbo-sacral spine	80.0 (24/30)		80.0 (12/15)		80.0 (12/15)		86.7 (26/30)		86.7 (13/15)		86.7 (13/15)		86.7 (26/30)		80.0 (12/15)		93.3 (14/15)
Knee joint	80.0 (24/30)		73.3 (11/15)		86.7 (13/15)		73.3 (22/30)		46.7 (7/15)		100.0 (15/15) ^a^		86.7 (26/30)		73.3 (11/15)		100 (15/15)
Shank	63.3 (19/30)		73.3 (11/15)		53.3 (8/15)		26.7 (8/30)		20.0 (3/15)		33.3 (5/15)		36.3 (11/30)		33.3 (5/15)		40.0 (6/15)
Thigh	60.0 (18/30)		60.0 (9/15)		60.0 (9/15)		20.0 (6/30)		20.0 (3/15)		20.0 (3/15)		16.6 (5/30)		13.3 (2/15)		20.0 (3/15)
Hip joint	66.7 (20/30)		60.0 (9/15)		73.3 (11/15)		46.7 (14/30)		33.3 (5/15)		60.0 (9/15)		33.3 (10/30)		46.7 (7/15)		20.0 (3/15)

^a^ 100% in the knee joint column for male modern dancers indicate that the whole group reported a serious injury in this area at least once during their career.

The instep and phalanges were not often affected by injuries in modern and folk dancers, though the prevalence in classical dancers was very high. In the group of classical dancers, 80% of the participants reported injuries of the metatarsus.

The lumbar spine was the most frequently injured area among the dancers. The prevalence of lumbar spine injury was similar in all dance styles and amounted to 80%. The knee joint was the second most commonly affected area, with >70% of modern and >80% of classical and folk dancers impacted. Female modern dancers reported a significantly lower prevalence of knee joint injury. The highest prevalence of hip joint injury was observed in classical dancers (66.7%), and a relatively high prevalence of hip joint injury was noted in modern dancers (60%). Meanwhile, the tibia and thigh were most affected by injuries in the group of classical dancers.

Based on the body diagram survey results, regardless of dance style, it was possible to identify the three principal body areas most affected by injuries: the lumbar spine, knee joint, and ankle joint.

**Figure 1:**
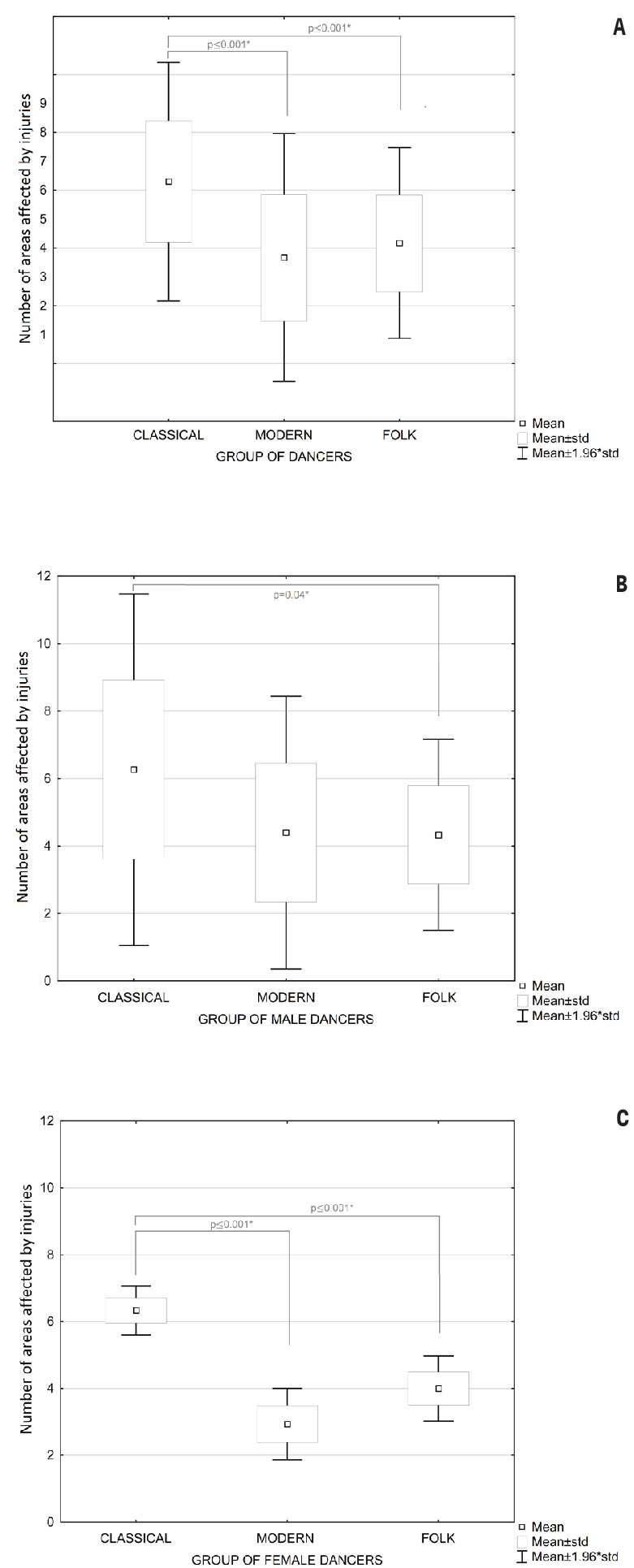
Number of areas affected by injuries in three groups of dancers (classical, modern, and folk) in relation to (A) the entire group, (B) male dancers, and (C) female dancers.

The mean number of areas affected by injuries was highest among the classical dancers (N=6.3). In the group of folk dancers, 4.17 areas were affected by injury (figure 1a). The indicator was similar among male and female dancers and across dance styles. Meanwhile, the lowest number of areas affected by injuries was found among modern dancers (3.67) and was 30% higher among male versus female modern dancers.

The ANOVA-based analysis revealed statistically significant differences between the three groups of dancers for the number of areas affected by injuries (F=14.622; P≤0.001*; η^2^=0.252; figure 1a), with Tukey’s post hoc test indicating a significant difference between classical and folk dancers (P≤0.001*) and between classical and modern dancers (P≤0.001*). However, no differences were found between modern and folk dancers in the number of injured areas (P=0.59).

Data analysis using one-way ANOVA highlighted statistically significant differences in the number of areas affected by injuries for female (F=13.204; P≤0.001*; η^2^=0,39) and male (F=4.039; P=0.025*; η^2^=0,16) dancers. Among the male dancers, the post hoc tests showed a difference between classical and folk dancers (P=0.04*; figure 1b). In turn, female classical and modern dancers (P≤0.001*) and female classical and folk dancers (P≤0.001*; figure 1c) differed in the number of areas affected by injury.

A weak, statistically significant, negative Spearman rank correlation was found between the number of areas affected by injuries and age when looking at the entire surveyed group of dancers surveyed (R=-0.285; P=0.007). Dividing the study group by sex and dance style did not show this correlation. There was no correlation between the number of areas affected by injuries and body weight. Spearman’s rank correlation test showed a weak negative correlation between the number of areas affected by injuries and the total experience for the entire group (R=-0.269; P=0.01) and the female group (R=-0.305; P=0.042). In the dancer male group and in the groups divided by sex, this relationship was not shown.

### Ground reaction forces generated during the performance of elements of three dance styles

Table 3 displays the maxGRF_z_, t_maxGRF_, and LR_GRFz_ results for the classical, modern, and folk dancers. The highest mean maxGRF_z_ values were obtained for jumps performed by classical dancers, which exceeded BW sevenfold. Jumps performed by modern dancers were characterized by a more than twofold lower impact (mean maxGRF_z_ = 3.35 BW) than those obtained by the classical dancers. Meanwhile, the mean value of maxGRFz exceeded BW fivefold in the folk dance group.

**Table 3:** Results of maxGRFz, t_maxGRF_, and LR_GRFz_ measurement for classical, modern, and folk dancers.

		maxGRFz [BW]	tmaxGRFz [s]	LRGRFz [BW/s]
CLASSICAL DANCE (group C)	Mean	7.33	0.06	131.63
	Std	1.31	0.02	52.66
	Median	7.25	0.06	122.31
	Min	5.31	0.04	50.57
	Max	11.11	0.11	264.52
	P-value (normal) Shapiro-Wilk test	0.006	0.028	0.166 ^a^
MODERN DANCE (group M)	Mean	3.35	0.38	51.17
	Std	1.16	0.32	77.76
	Median	3.07	0.42	7.89
	Min	1.7	0.01	2.25
	Max	6.39	1.17	333.33
	P-value (normal) Shapiro-Wilk test	<0.001	<0.001	<0.001
FOLK DANCE (group F)	Mean	5.1	0.08	92.6
	Std	1.72	0.04	65.86
	Median	5.21	0.07	76.03
	Min	1.59	0.02	8.43
	Max	8.4	0.23	278.5
	P-value (normal) Shapiro-Wilk test	0.776 ^a^	<0.001	<0.001

^a^ Normal distribution with P≥0.05

In classical dance, the highest maxGRF_z_ values were obtained for grand jeté: 9.18 (1.44) BW and grand pas de chat: 7.89 (1.03) BW, in modern dance for grand jeté en tournant: 4.67 (1.59) BW and travelling leap: 5.13 (1.53) BW, and in folk dance for Przeskok przez ciupagę: 7.23 (2) BW and Skoki w sarenkach: 6.63 (0.4) BW (see supplementary material, URL).

Statistically significant differences in maxGRF_z_ values were found between males and females in the group of dancers who participated in the post-jump impact force measurements (t=3.729; P<0.01*, d=0.559).

Landing after an jumps in modern dance was characterized by an approximately five times longer time between the moment of first foot contact with the ground to the moment of reaching maxGRF_z_ (mean t_maxGRFz_ = 0.38 s), which was significantly greater than the classical and folk dancers. In addition, the modern dancers had the lowest mean and median values for LR_GRFz_, while the classical dancers had the highest LR_GRFz_ values.

The Kruskal-Wallis analysis revealed statistically significant differences between the three groups of dancers for all GRF parameters measured (P≤0.001*), with the subsequent post hoc tests (Dunn-Bonferroni) identifying differences between all groups of dancers for all parameters, including maxGRFz (χ^2^ (2)=66.348; P≤0.003*; ε^2^=0.486), tmaxGRF (χ^2^ (2)=45.239; P≤0.001*; ε^2^=0.156), and LRGRFz (χ^2^ (2)=54.449; P≤0.001*; ε^2^=0.274),

### Comparison of survey results and ground reaction force measurements

**Figure 2:**
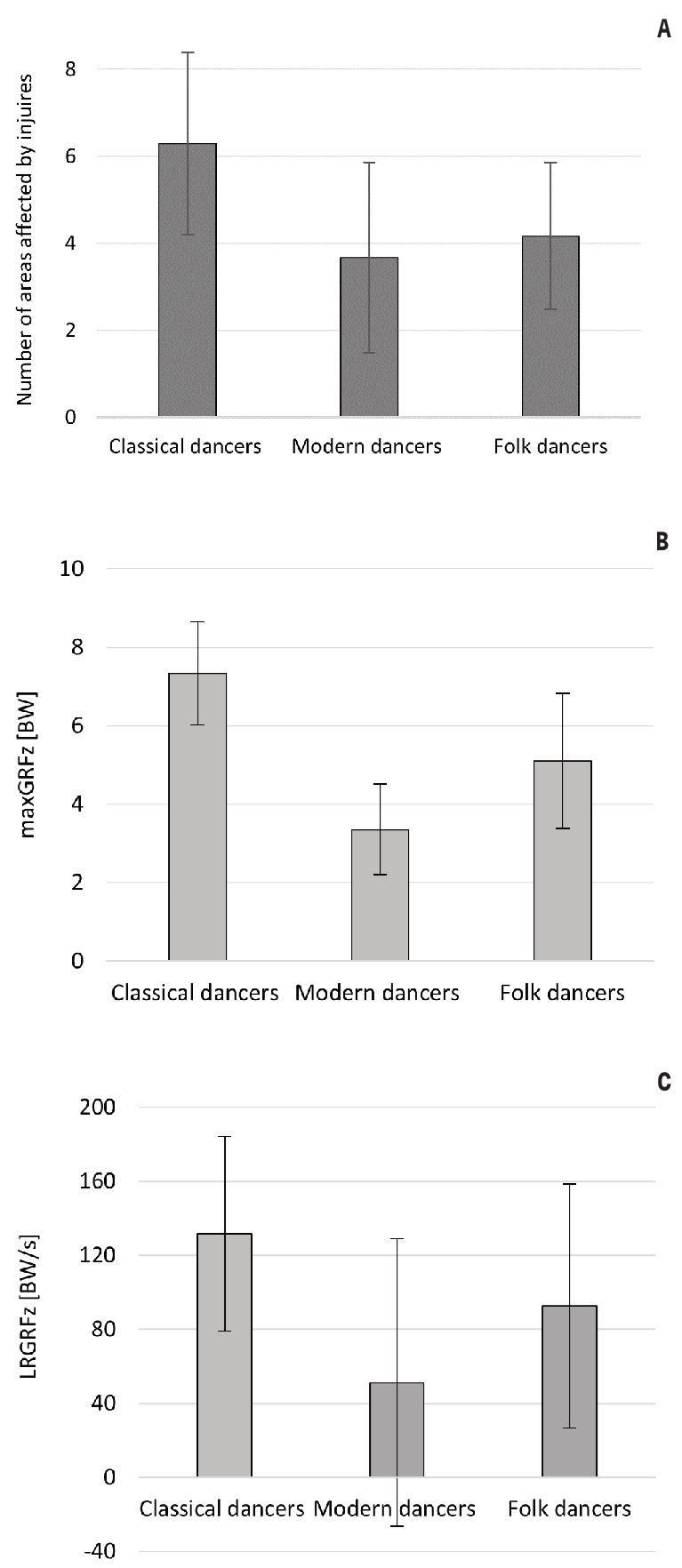
Comparison of selected survey and ground reaction force measurement results for (A) number of injured areas, (B) maxGRFz, and (C) LRGRFz.

Figure 2 shows a comparison of the number of injured areas with the GRF measurement results, which demonstrates that the classical dancers had the most injured areas, and the modern dancers had the least. In addition, the classical dancers had the highest maxGRF_z_ and LR_GRFz_ values, and the modern dancers had the lowest values.

## Discussion

The primary observations resulting from the tests are the following: (i) the mean number of areas affected by injuries that temporarily precluded further dance was the highest among classical dancers; (ii) there were statistically significant differences between the three groups of dancers for all GRF parameters, with the highest maxGRF_z_ values obtained for jumps performed by classical dancers; and (iii) the lumbar spine, knee joint, and ankle joint were the most affected body areas in all three groups of dancers. The discussion below presents several possible explanations and interpretations of the findings.

### Classical dancers as the group at highest risk of sustaining injuries?

The number of injured areas was highest among the classical dancers and amounted to 6.3, which is a very high index given the age of the study group members [mean 23.3 (SD 3.7) years], although previous works investigating this professional group found a similar outcome (18, 23). These findings also indicated that choreographic elements performed by classical dancers generated the highest maxGRF_z_, which was 7.33 times BW. The highest maxGRF_z_ values were obtained for the grand jeté with 9.18 (1.44) BW and the grand pas de chat with 7.89 (1.03) BW.

GRF values are not the only factor responsible for high strains on a dancer’s motor system. In classical dance, the time to obtain maxGRF is very short, whereas the index of force growth is high, which was evident in this study and previous work (19). These characteristics distinguish the classical dance style from the remaining dance styles. The necessity for sufficient shock absorption after a very short time (t_maxGRFz_ = 0.06 (0.02) s) is a very specific element of classical dance and a probable factor in injuries to the locomotor system elements critical for shock absorption, such as the foot and knee (6, 20). In addition, the classical dancer works in ballet pointé shoes, and most injuries of the ankle joint and the foot are connected with performing pointé and demi pointé in classical dance (25), which are the most characteristic elements of classical dance and require multi-annual exercises to perform.

According to most authors investigating dancers’ feet, the muscles responsible for the foot and ankle joint should be able to sustain appropriately high force to perform pointé-work (26, 27). The introduction of pointé-work-related exercises before the dancer’s feet obtain their functional maturity weakens the feet and increases their susceptibility to injuries (27). Plantar flexion of 180º in relation to the central axis of the tibial bone and foot is a pre-condition of proper, entire, and safe pointé-work (26, 27). However, the combination of high impact loads, defined as the level of maxGRF_z_ (5.31–11.11 BW for the analyzed jumps), and weakness caused by the premature introduction of pointé-work in technical exercises of classical dance increase injury risk and leads to a high percentage of metatarsus (80%) and ankle joint (83.3%) injuries.

### The injury triad of professional dancers – spine, knee, and ankle

The statistical tests performed showed statistically significant differences in maxGRF_z_ and the number of areas affected by injuries between the three dancing groups. Among classical dancers, the number of areas affected by injuries was more than twice that of modern dancers and more than that of folk dancers. The same relationship was found for the maxGRF_z_ values, which were almost double for classical dancers compared to modern dancers and more than 1.5 times for classical dancers compared to folk dancers (figure 2, table 3).

Irrespective of the dance style, the prevailing body areas indicated by the dancers as most susceptible to injuries were the spine, knee joint, and ankle joint (table 2). Spine injuries were indicated by 80–93% of all dancers. More than half of all surveyed dancers (from 46% to 100%) suffered one serious knee joint injury in their career, and as many as 93% of female classical dancers declared that the ankle joint was the most commonly injured area of the musculoskeletal system (79% of them also listed the foot). The unquestionable “winner” in this inglorious ranking is the spine.

According to Solomon et al (3), 60–80% of all professional dancers experience back pain or spine injuries during their careers, with the lower back being the most frequently affected area. Back pain represents one of the most common diseases across various populations of workers worldwide (28, 29). The lumbar spine connects to the pelvis at the L_5_-S_1_ joint, and this area is usually the source of pain and dysfunction among dancers (8–10, 30, 31). Lower back pain (LBP) is an ongoing injury among dancers that has a lasting negative effect, even after they have stopped dancing professionally (31). Densitometric tests by Gorwa et al (20) revealed that nearly all the dancers participating in their study (N=44) suffered from degenerative changes in the lumbar spine. Undoubtedly, such changes are due to the requirements related to classical dance, such as the emphasis on the high suppleness of this part of the body and the transfer of high GRF.

Raising the heels during choreographically demanding elements of classical dance (relevé on pointé, demi-pointé relevé, and arabesque) or while folk dancing in high heels deepens lordosis and reduces lumbar spine mobility in the sagittal plane (32). Deepened lumbar lordosis generates very high shear forces on intervertebral discs and posterior parts of vertebrae (32). In the landing phase following a jump, the spine constitutes the distal part of the biokinetic chain, regardless of dance style, and individual components of the chain dampen GRF. However, in cases of improper shock absorption (16, 33), inappropriate ground (34), or defective footwear (25), it is the spine that absorbs the ultimate (and even higher) GRF. Other harmful factors include mistakes made when partnering (32) and frequent traveling (touring) (35, 36).

Professional dancers frequently suffer from knee joint injuries, which constitute 7–29% of all injuries in this group (11–14, 37). According to the results of the current study, the prevalence of knee joint injury amounts to >70% among modern dancers and >80% among classical and folk dancers (table 2). Male classical and modern dancers indicated the knee joint as the dominant injury accompanying their profession. In turn, the prevalence of knee joint injuries was >70% among female classical and folk dancers and 47% among modern dancers. It is extremely important that the lower limb is properly positioned at the knee joint during landing to absorb the impact forces. Gorwa et al (38) indicate that after a jump in modern dance, the torque at the knee joint can be 3–4 times higher than the torque at the ankle joint.

The patellofemoral joint is often the source of pain and dysfunction among professional dancers (6–10), whose daily training includes hundreds of pliés, relevés, and jumps, in addition to preparation for performances. Such excessive overtraining of extensor mechanisms may lead to patellofemoral syndrome, patellar tendinitis, or patellar tendon enthesopathy (6). Also, dancers’ posterior femoral muscles (muscles of the hamstring group) remain tense (39) as a necessity for proper shock absorption, and the continuous load affecting the extensor mechanism makes the knee joint one of the most loaded joints.

This study showed statistically significant differences in maxGRF_z_ levels by sex (P<0.01*). Male dancers are more at risk of sustaining serious knee joint injuries than female dancers (40). Such a fact could result from varying sex-related BW, yet the most logical explanation points to the different kinetic tasks performed by male and female dancers, particularly dynamic tasks such as jumps (41). The roles of the male dancers in Polish folk dance are also very different. They are dominated by bravura jumps (as in the highland dances), and low positions (such as dancing in a squatting position or “przeskok przez ciupagę”), which require the involvement of the knee joint. However, this difference disappears in modern dance, where men and women dance similar elements (21, 42). Nevertheless, it is possible to notice differences in expression and manner of performance during a show, particularly during jumps. Females, being lighter, appear more delicate than males, who jump with bravado. In this dance style, knee joints are positioned very low, the so-called deep plié. Such a posture generates high tension resulting from improperly positioned knee joints in relation to the position of ankle joints (43). Enormous forces generated may lead to the partial tear of large ligaments or even meniscus fracture (2, 15, 17, 44). Importantly, today’s artists often dance on a hard floor that does not absorb shocks during falls or landings (33, 42).

During the performance of a dancing jump, the vertical GRF component reaches a value a few times BW in the landing phase (19–21, 43), which applies to all styles discussed in this study. In this experiment, the highest maxGRF_z_ was achieved for the following types of jumps: grand jeté with 9.18 (1.44) BW in classical dance, travelling leap with 5.13 (1.53) BW in modern dance and Przeskok przez ciupagę with 7.23 (2) BW in folk dance.

According to Weiss et al (26), muscles responsible for the foot and ankle joints must be sufficiently strong before commencing climbing on toes training. Introducing these exercises before reaching the full functional maturity of the foot weakens it and increases its susceptibility to injuries. It should be noted that the foot and the ankle joint are the first links in the bio-kinematic chain that are involved in the landing phase and that they take on the highest value of maxGRF_z_. This is evident in the topography of the injury – classical and folk dancers, in whom the highest .maxGRF_z_ values were measured [classical dancers: 7.33 (SD 1.31) BW, folk dancers: 5.1 (SD 1.72) BW], indicate that these areas are among the most prone to injury. Working in pointé and demi-pointé positions also undoubtedly puts considerable loads on the ankle joints. In the current study, females specializing in classical dance listed ankle joints (93%) and the metatarsus (80%) as the locations most susceptible to injuries, while it was the least injured area in female modern dancers. The prevalence of ankle joint and metatarsus injuries was also very high among classical dancers and amounted to 73% and 80%, respectively. Folk dancers had a high prevalence of ankle joint injuries (83%) but relatively low metatarsus injuries (16.7%). It would be worthwhile analyzing the practice of these three groups of dancers to find the reasons behind this. A female classical dancer works on pointé, depending on the type of performance, for more than half of her working day, whereas female modern and folk dancers only do so during lessons (classical warm-up). Furthermore, modern and folk dancers often dance on a hard floor (especially during tours) that does not absorb falls and landings, and they use a sub-optimal warm-up system, in their own opinion.

### Limitations and directions for future research

One of the limitations of this work is that the survey forms are subjective and have a limited degree of accuracy. Future research requires a larger number of dancers of individual styles and the seasonal monitoring of injuries in distinct dance groups. This study involved the analysis of the vertical variable of GRF, whereas subsequent stages will include the association of the resultant GRF values with movement kinematics, including the positioning of individual body segments in space. The authors realize that the GRF level is not the only factor affecting the topography of injuries in professional dancers. Other factors include the position of individual body segments in space, shock absorption-related mistakes, repeated kinetic schemes, biomaterial fatigue, over-exercise, improper diet, types of shoes and stage/floor materials (19–21).

### Concluding remarks

Regardless of dance style (classical, modern, and folk dance) and sex, the lower spine and the knee joint were the body areas most affected by injuries in professional dancers. The prevalence of lumbar spine injuries is dominant in all styles of dance.

Classical dance is also characterized by injuries to the ankle joints, instep, phalanges, and hip joints. The susceptibility of the instep and phalanges was particularly determined by classical dance and was less common in modern and folk dancers.

The level of the maxGRF_z_ variable in relation to typical jumps in classical, modern, and folk dance was significantly different (P<0.01*). The highest mean values were observed in jumps performed by classical dancers, which exceeded BW sevenfold. Individual dance styles also significantly varied in terms of t_maxGRF_ and LR_GRFz_. There were statistically significant differences in the maxGRF_z_ values according to sex (P<0.01*), but no differences according to age and total experience.

The highest maxGRF_z_ values were obtained for the following jumps: grand jeté and grand pas de chat in classical dance, grand jeté en tournant and travelling leap in modern dance, and Przeskok przez ciupagę and Skoki w sarenkach in folk dance.

The sum of injury-affected areas differed significantly across the various dance styles and was connected with the impact forces transferred by the dancer’s musculoskeletal system.

## Supplementary material

Supplementary material
