# Supplementary material for: Ground reaction force as a factor responsible for the topography of injuries in professional dance. An analysis of three dance styles: classical dance, modern dance, and folk dance
Source: Scand J Work Environ Health. 2024 Feb 28;50(2):103–12. doi: 10.5271/sjweh.4137 (PMC10928442; doi:10.5271/sjweh.4137)
Supplement: Supplementary material [file SJWEH-50-103-S001.pdf]

Ground reaction force as a factor responsible for the topography of injuries in professional dance. An analysis of three dance styles: classical dance, modern dance, and folk dance[1]

by Joanna Gorwa, PhD, DSc,[2] Katarzyna Nowakowska-Lipiec, PhD, Robert Michnik, PhD, DSc

1. Supplementary material

2. Correspondence to: Joanna Gorwa, Department of Biomechanics, Faculty of Sport Sciences, Poznan University of Physical Education, Królowej Jadwigi 27/39, 61-871 Poznań, Poland. [E-mail: gorwa@awf.poznan.pl]

The mean maxGRF values and standard deviations obtained for style-specific jumps:

- **Classical dance:**
  - Grand pas de chat: 7.89 (1.03) BW,
  - Grand pas assemble: 6.95 (0.27) BW,
  - Entrelace: 6.87 (0.80) BW,
  - Grand jete: 9.18 (1.44) BW,
  - Saut de basque: 5.55 (0.11) BW,
  - Pas jete: 5.31 (0.33) BW,
  - Turn: 7.63 (0.22) BW,
  - Jete en tournant: 7.12 (0.08) BW,
  - Ballonne: 7.54 (0.14) BW,
  - Pas echappe: 7.12 (0.55) BW,
  - Sissonne ouverte: 6.21 (0.18) BW,
  - Jete passe: 7.02 (0.14) BW,
  - Pass de poisson: 6.13 (0.36) BW.
- **Modern dance:**
  - Stag jump: 3.02 (0.99) BW,
  - Grand jete modern: 3.88 (1.73) BW,
  - Skip with throwing leg to the side: 4.60 (0.16) BW,
  - Travelling leap: 5.13 (1.53) BW,
  - Jump with "ront" & twist: 3.49 (0.47) BW,
  - Skip with forward leg throw: 3.74 (0.89) BW,
  - Grand jeté en tournant: 4.67 (1.61) BW,
  - Skip: 3.88 (1.73) BW,
  - Leap: 3.12 (0.73) BW,
  - Sisonne overt parallel: 2.84 (0.98) BW,
  - Flat pas de chat: 3.65 (1.52) BW,
  - Vertical jump by Ewa Wycichowska: 3.34 (0.04) BW,
  - Vertical jump head tilted back: 3.85 (0.57) BW,
  - Half stag jump: 3.02 (1.39) BW,
  - Wide open legs jump to the side: 3.74 (1.01) BW,
  - Flick-jeté jump: 4.08 (1.40) BW,
  - Flick-jeté leap: 3.69 (0.96) BW.

- **Folk dance (Polish folk dances, the proper names of the analysed dance jumps are given):**
    - ‘Podcinane – Śmigło’: 6.29 (0.09) BW,
    - ‘Miotły’: 1.98 (0.05) BW,
    - ‘Skoki w obrocie (kołomajki)’ : 5.95 (0.09) BW,
    - ‘Skoki w sarenkach’: 6.63 (0.40) BW,
    - ‘Łamańce-tańce góralskie’: 3.30 (0.08) BW,
    - ‘Podskok przez nogę’: 4.65 (0.42) BW,
    - ‘Przeskok przez ciupagę’: 7.23 (2.00) BW,
    - ‘Araby’: 5.60 (0.04) BW,
    - ‘Kabriol Krakowski’: 4.05 (0.15) BW,
    - ‘Roznóżki’: 4.05 (0.42) BW,
    - ‘Wyrzut skoki góralskie’: 6.40 (1.29) BW,
    - ‘Kołomajki obroty’: 5.74 (0.02) BW,
    - ‘Kabriol krakowski’: 4.05 (0.15) BW.
